# Complete Genome Sequence of *Moraxella osloensis* Strain KMC41, a Producer of 4-Methyl-3-Hexenoic Acid, a Major Malodor Compound in Laundry

**DOI:** 10.1128/genomeA.00705-16

**Published:** 2016-07-21

**Authors:** Takatsugu Goto, Hideki Hirakawa, Yuji Morita, Junko Tomida, Jun Sato, Yuta Matsumura, Asako Mitani, Yu Niwano, Kohei Takeuchi, Hiromi Kubota, Yoshiaki Kawamura

**Affiliations:** a Division of Anaerobe Research, Life Science Research Center, Gifu University, Gifu, Japan; b Department of Technology Development, Kazusa DNA Research Institute, Chiba, Japan; c Department of Microbiology, School of Pharmacy, Aichi Gakuin University, Aichi, Japan; d R&D Safety Science Research, Kao Corporation, Tochigi, Japan; e R&D Household Products Research, Kao Corporation, Wakayama, Japan; f R&D Perfumery Development Research, Kao Corporation, Tokyo, Japan

## Abstract

We report the complete genome sequence of *Moraxella osloensis* strain KMC41, isolated from laundry with malodor. The KMC41 genome comprises a 2,445,556-bp chromosome and three plasmids. A fatty acid desaturase and at least four β-oxidation-related genes putatively associated with 4-methyl-3-hexenoic acid generation were detected in the KMC41 chromosome.

## GENOME ANNOUNCEMENT

*Moraxella osloensis* exists in various environmental sources (1) and rarely causes opportunistic infections such as bacteremia and meningitis (2). It is frequently detected in laundry after washing and has the potential to generate 4-methyl-3-hexenoic acid (4M3H), a major malodor compound in laundry that has a “wet-and-dirty-dustcloth-like” odor (1, 3). Little is known about the virulence and odor-generating mechanisms of *M. osloensis*.

Here, we report the complete genome sequence of *M. osloensis* strain KMC41 (PAGU 1681), isolated from laundry with malodor (1). Genomic DNA was extracted and sequenced using the Roche 454 GS FLX Titanium (8-kb paired-end library) and Roche 454 GS FLX++ (single-end library) platforms. After filtering the raw reads (Phred-equivalent score ≥10), the remaining 333,649 reads, with about 38.4-fold genome coverage, were assembled using Newbler version 2.6, generating 81 scaffolds with an *N*_50_ length of 56,102 bp. *In silico* gap closure using the MIRA version 3.0 assembler, including manual editing, generated 14 scaffolds; the remaining gaps among the scaffolds were completely closed by sequencing the PCR products spanning the gaps. Protein-coding sequences (CDSs), tRNA genes, and clustered regularly interspaced short palindromic repeats (CRISPRs) were predicted using MetaGeneAnnotator (4), tRNAscan-SE version 1.23 (5), and CRISPRFinder (6), respectively. Functions of all of the CDSs were manually annotated based on the results of BLASTx searches against the NCBI NR database (7). Orthologous genes between KMC41 and *Escherichia coli* strain K-12 (MG1655) were detected by Mauve (8) and BLASTn (7) analyses.

The KMC41 genome comprises a 2,445,556-bp chromosome (G+C content: 43.9%) and three plasmids (pMOSL1, pMOSL2, and pMOSL3, comprising 76,124, 62,260, and 37,064 bp, respectively). The chromosome contains 2,186 predicted CDSs, 47 tRNA genes, four rRNA operons, and one CRISPR. The pMOSL1, pMOSL2, and pMOSL3 plasmids encode 73, 67, and 45 CDSs, respectively. PHAST (9) did not detect any prophage region in the genome.

A reciprocal best-hit analysis of CDSs between KMC41 and *Moraxella catarrhalis* strain BBH18 (10) using BLASTp (7) (*E* value cutoff: 10^−20^) showed that 48.4% of the CDSs from KMC41 were in orthologous relationship with the CDSs from BBH18. The KMC41 genome encoded only two homologs (*ompE*, *ompCD*) of the eight major virulence factors (10) reported in BBH18. This result concurs with the epidemiological data that *M. catarrhalis* is a major pathogen in the respiratory tract, whereas *M. osloensis* rarely causes infections.

Judging from its structure, 4M3H may be derived from branched-chain fatty acids in sebum on laundry by desaturation and β-oxidation reactions. The KMC41 genome encodes a Δ^9^-fatty acid desaturase gene (*des* encoded by MOSL_0712), and at least four genes (*fadA*, *fadB*, *fadD*, and *fadH* encoded by MOSL_1738, MOSL_1739, MOSL_1881, and MOSL_1862, respectively) associated with β-oxidation. Indeed, quantitative PCR analysis revealed that the expression of MOSL_0712 and MOSL_1739 were upregulated during 4M3H generation (unpublished data). Further studies about metabolic pathways and gene functional analyses will help clarify the malodor-generating mechanisms in *M. osloensis*.

### Nucleotide sequence accession numbers.

The *M. osloensis* strain KMC41 genome sequence was deposited in DDBJ/ENA/GenBank under the accession numbers AP017381 (chromosome) and AP017382 to AP017384 (plasmids).
